# Supplementary material for: WordCommentsAnalyzer: A windows software tool for qualitative research
Source: F1000Res. 2018 Sep 4;7:536. Originally published 2018 May 3. [Version 2] doi: 10.12688/f1000research.14819.2 (PMC6137411; doi:10.12688/f1000research.14819.2)
Supplement: Supplementary file 3 [file f1000research-7-17614-s0002.tgz › b2a79496-4ada-47c4-9682-a99ce867836d.zip!/Eight Iranian Health Journals/Journal of Health System Research _15.docx]

© Journal of Health System Research . This work is licensed under CC BY-NC 4.0 (https://creativecommons.org/licenses/by-nc/4.0/).

Volume & Issue: دوره 10، شماره 3، 1393

Title: نقش الگوی غذایی برهایپرگلایسمی ومقاومت به انسولین: یک مرور سیستماتیک

http://hsr.mui.ac.ir/index.php/jhsr/article/view/1439

چکیده مقدمه : مطالعه حاضر به منظور بررسی سیستماتیک الگوهای غذایی غربی و تأثیر آن‌ها در بروز مقاومت به انسولین و نوع دوم بیماری دیابت ملیتوس می‌باشد. روش ها: این بررسی با مرور موتورهای جستجو از قبیل : ISI, Cochrane Central Register of Controlled Trials databases, Pubmed, Iran Medex and MagIran انجام شد. لغات کلیدی مورد جستجو مشتمل بر: "دیابت ملیتوس"، "سندرم متابولیک"، "مقاومت به انسولین"، "الگوی غذایی" بود.  یافته‌ها: جستجوی انجام شده در محدوده زمانی سال‌های 2011-1992 و به صورت جامع در قالب مجلات، کتاب‌های الکترونیکی، سمینارها و سمپوزیوم‌ها اجرا شد. انواع مطالعات اعم از مطالعات کوهورت آینده‌نگر، کارآزمایی بالینی و مقطعی براساس بررسی عنوان ، خلاصه و متن کامل مورد ارزیابی قرار گرفته و پس از حذف مطالعات دارای گروه هدف حیوانی، کودکان و نوجوانان از مجموعه، 738 مورد استخراج شد. نتایج حاکی از تأثیرمعنی‌دارالگوی غذایی "غربی" غنی از گوشت قرمز، غذاهای فرایندشده و سرخ کرده، غلات تصفیه شده و دارای نمایه گلایسمی بالا در بروز هایپرگلایسمی است. نتیجه‌گیری: تبعیت از عادات غذایی ناسالم و سرشار از اسیدهای چرب ترانس و اسیدهای چرب اشباع ، کربوهیدرات‌های ساده و تصفیه شده با نمایه گلایسمی بالا با ایجاد مقاومت به انسولین، هایپرگلایسمی و خطر دیابت نوع 2 مرتبط است.

Title: آنتی اکسیدان های رژیم غذایی و بیماری پریودنتال

http://hsr.mui.ac.ir/index.php/jhsr/article/view/1980

مقدمه: بیماری پریودنتال یکی از بیماری‌های رایج دهانی است که استرس اکسیداتیو نقش اساسی در پاتوژنز این بیماری ایفا می‌کند. شروع و پیشرفت این بیماری به تعادل دقیق بین بیوفیلم‌های میکروبی دهانی و پاسخ میزبان بستگی دارد. یکی ازمهم‌ترین عوامل در ابتلا، پیشگیری و درمان این بیماری تغذیه می‌باشد. با توجه به نقش استرس اکسیداتیو در پاتوژنز بیماری پریودنتال و کاهش ظرفیت تام آنتی‌اکسیدانی بدن در بیماران پریودنتال، توصیه به مصرف مناسب از مواد غذایی حاوی آنتی‌اکسیدان، می‌تواند در درمان این بیماران مفید واقع شود. هدف از این مطالعه مروری، بررسی نقش آنتی‌اکسیدان‌های رژیم غذایی، در بیماری پریودنتال می‌باشد. روش‌ها: کلیه مقالات مرتبط مورد استفاده در این مطالعه از پایگاه اطلاعاتی Pubmed و Google Scholar و Iranmedex ، که بین سال‌های 1991 تا 2013 منتشر شده بودند، جستجو شد و مورد مطالعه و بررسی قرار گرفت. پس از مطالعه خلاصه 60 مقاله به‌دست آمده، متن کامل 30 مقاله مورد بررسی قرار گرفت. یافته‌ها: مطالعات بررسی شده نشان دادند که آنتی‌اکسیدان‌های رژیم غذایی مانند ویتامین ,C ویتامین ,E کارتنوئیدها (α و β کاروتن، β کریپتوگزانتین، زئوزانتین و لیکوپن) و پلی‌فنل‌ها (پروآنتوسیانیدین و فلاونوئید‌ها) در جلوگیری از استرس اکسیداتیو و محافظت در برابر بیماری پریودنتال نقش بسزایی دارند. نتیجه‌گیری: بر اساس یافته‌های این مطالعه مروری می‌توان نتیجه گرفت که، آنتی‌اکسیدان‌های رژیم غذایی می‌تواند در پیشگیری و درمان بیماران پریودنتیت مفید واقع شوند.

Title: بررسي وضعیت فعالیت فیزیکی در بیماران ديابتی نوع 2 براساس سازه های الگوی مراحل تغییر رفتار

http://hsr.mui.ac.ir/index.php/jhsr/article/view/1323

مقدمه: دیابت یکی از مشکلات اصلی سیستم بهداشتی در جهان است و فعالیت بدنی به‌عنوان یکی از فاکتورهای قابل تعدیل و اصلی سبک زندگی در مدیریت و کنترل آن محسوب می شود. این مطالعه با هدف تعیین وضعیت فعالیت بدنی در بیماران دیابتی نوع 2 در شهرستان خرم آباد بر اساس الگوی مراحل تغییر انجام شد. روش‌ها: جامعه آماری این مطالعه شامل بیماران دیابت نوع 2 تشخیص داده شده در شهرستان خرم آباد در سال 91 می‌باشد. که به روش نمونه‌گیری آسان تعداد 393 نفر وارد مطالعه گردید. با مراجعه به مراکز بیماران دیابتی نوع 2 و پس از توضیح اهداف مطالعه و اخذ رضایت ‌نامه برای شرکت در مطالعه، پرسش‌نامه بین بیماران توزیع و اطلاعات جمع‌آوری شد. داده‌های مطالعه از طریق پرسش‌نامه 6 قسمتی جمع‌آوری گردید. داده‌ها با استفاده از نرم‌افزار آماری SPSS-18 و آزمون‌های ضریب همبستگی پیرسون، کای‌دو، تی مستقل و آزمون آنالیز واریانس با در نظر گرفتن سطح معنی‌داری کمتر از 5 درصد تجزیه تحلیل گردید. یافته‌ها: 9/48% شرکت‌کنندگان در مرحله پیش تفکر و تنها 15% افراد در مرحله نگهداری بودند. میانگین ورزش در روز 6/20±9/11 دقیقه بود که میانگین آن در گروه نگهداری بطور معنی‌داری بیشتر از بقیه گروه‌ها بود ارتباط معنی‌دار معکوسی بین میانگین ورزش در روز با منافع درک شده و ارتباط مستقیم معنی‌داری با فرایندهای تغییر، خودکارامدی و تعادل تصمیم‌گیری وجود داشت. تعداد دفعات ورزش در هفته بیماران با میانگین خودکارامدی ارتباط مستقیم معنی‌داری داشت (001/0P<). نتیجه‌گیری: اکثریت بیماران برنامه‌ ای برای فعالیت بدنی در 6 ماه آینده نداشتند و سطح فعالیت بدنی بیماران نامطلوب بود. با توجه به ارتباط خودکارامدی و تعادل تصمیم گیری با میانگین ورزش در روز و دفعات ورزش، تدارک برنامه‌ های آموزشی با تمرکز روی این سازه‌ها می‌تواند به بهبود سطح فعالیت بدنی کمک کند

Title: : بررسی رابطه شاخص‌های استرین فیزیولوژیکی (PSI ) و شاخص دمای تر گویسان(WBGT ) در زنان در اتاقک شرایط جوی

http://hsr.mui.ac.ir/index.php/jhsr/article/view/1618

مقدمه: نیاز به ارزیابی استرس ناشی از مواجهه با گرما به عنوان یکی از عوامل زیان‌آور در صنعت جهت کنترل مؤثر آن ضروری می‌باشد. هدف از انجام این مطالعه بررسی رابطه شاخص‌های استرین فیزیولوژیکی (PSI یا physiological strain index) و شاخص دمای تر گویسان (WBGT یا Wet Bulb Globe Temperature Index) در زنان در اتاقک شرایط جوی بود. روش‌ها: این مطالعه تجربی،بر روی 36 نفر از دانشجویان زن سالم در شرایط جوی کنترل شده در گستره دمایی C 32-22 از شاخص WBGT در حالت نشسته طی 2 ساعت انجام گرفت. پارامترهای دمای دهانی و ضربان قلب جهت محاسبه شاخص  PSIبه فاصله هر 5 دقیقه اندازه‌گیری شد. داده‌ها با آماره‌های توصیفی وآزمون‌های رگرسیون و همبستگی تحلیل گردیدند. یافته‌ها: میانگین ضربان قلب و دمای دهانی در مواجهه با گرما bpm 91/87 و C 89/36 به‌دست آمد. مقدار حداقل و حداکثر شاخص دمای تر گویسان به ترتیب 49/25 وC 32/1 ،حداقل و حداکثر ضربان قلب 01/83 و 97/90 bpm و حداقل و حداکثر دمای دهانی7/36 ،C 01/37 به دست آمد. بین شاخص WBGT و شاخص PSI ( 001/0, P< 71/0( r=، دمای دهانی ( 001/0, P< 98/0 ( r= و ضربان قلب ( 001/0 ,P< 77/0( r= رابطه مستقیم و معنی‌داری وجود دارد ( 05/0( P< . نتیجه‌گیری: نتایج این مطالعه نشان داد که شاخص WBGT با متغیرهای فیزیولوژیک ضربان قلب، دمای دهانی و شاخص PSI در گستره دمایی°C 32-22 و رطوبت40% در زنان با پوشش اسلامی رابطه مستقیم ومعنی‌داری وجود دارد. همزمان با افزایشWBGT ضربان قلب،دمای دهانی و شاخص PSI نیز افزایش یافته است.

Title: اثر بسته‌بندي تحت اتمسفر اصلاح شده بر خصوصيات فيزيکي و شميايي دانه‌هاي انار طی نگهداری در دماهای متفاوت

http://hsr.mui.ac.ir/index.php/jhsr/article/view/1400

مقدمه: فراوري انار به صورت خشک کردن، رب، آب‌گيري و کنسانتره کردن باعث کاهش خصوصيات تغذيه‌اي و از بين رفتن عطر و طعم ويژه آن مي‌گردد. از طرف ديگر مشکل جدا کردن پوسته از دانه، تازه خوري آن را محدود مي‌نمايد به همين دليل توليد دانه‌هاي آماده مصرف امکان مصرف تازه آن را آسان‌تر مي‌کند. در اين مطالعه، تغييرات خصوصيات کيفي دانه‌هاي انار آماده مصرف تحت بسته‌بندي اتمسفر اصلاح شده مورد مطالعه قرار گرفت. روش‌ها: دانه‌ها در فيلم پلي‌اتيلن با دانسیته کم با ضخامت 100 ميکرومتر بسته‌بندي گرديد و به ترتيب به مدت 11 و 9 روز در دماي 4 و 10 درجه سانتي‌گراد ذخيره شدند. در طي نگهداري تغييرات غلظت گاز و خصوصيات کيفي شامل اسيديته، pH، مواد جامد محلول، بافت و رنگ تعيين گرديد. یافته‌ها: طي نگهداري، غلظت اکسيژن در داخل بسته از % 9/20 به %0 درصد و اسيديته دانه‌هاي انار از %25/1 به %07/1 کاهش و غلظت دي‌اکسيدکربن از %03/0 به %7/20 افزايش مي‌يابد. وزن دانه‌ها در بسته و دما اثر معني‌داري بر شدت تغييرات غلظت هر دو گاز نشان داد. ميزان pH دانه‌هاي انار در روزهاي اول نگهداري افزايش ولي در ادامه کاهش يافت. ميزان مواد جامد محلول، بافت و رنگ دانه‌هاي انار طي نگهداري تغيير معني‌داري نداشت. نتیجه‌گیری: دانه‌هاي انار در بسته‌هاي پلي‌اتيلني و دماي نگهداري 4 و 10 درجه‌سانتي‌گراد به ترتيب 7 و 5 روز قابل نگهداري است. احتمالا در دماهاي بالاتر به منظور جلوگيري از رسيدن به غلظت‌هاي بحراني اکسيژن و دي اکسيدکربن ايجاد سوراخ‌هايي در فيلم بسته‌بندي ضروري به نظر مي‌رسد.

Title: اثر مدت پیش تخمیر و نگهداری به‌صورت منجمد بر کيفيت خمير سنگک منجمد و نان حاصل از آن

http://hsr.mui.ac.ir/index.php/jhsr/article/view/1412

مقدمه: نان تازه داراي زمان ماندگاري كوتاهي است و مقبوليت آن نزد مصرف‌كننده با افزايش فاصله زماني بين پخت تا مصرف به نحو چشمگيري كاهش مي‌يابد. يكي از راه‌هاي مقابله با مشكل مذكور استفاده از تكنولو‌ژي خمير منجمد مي‌باشد. از اين رو در اين تحقيق اثر پيش تخمير و نگهداري منجمد بر كيفيت خمير و نان سنگك حاصل از آن مورد بررسي قرار گرفت. روش‌ها: نمونه‌ها در سه حالت تخمیر نشده، پیش تخمیر شده بدون فرم‌دهی و پیش تخمیر شده فرم‌دهی شده تهیه شدند. پیش تخمیر برای همه نمونه‌ها 30 دقیقه اعمال شد. سپس خمیرها در دماي 25- درجه سانتي‌گراد در فريزر جريان هواي سرد منجمد شده و به مدت 24 ساعت، 4، 7، 10 و 13 هفته در فریزر 18- درجه سانتی‌گراد نگهداری شدند.. بررسي کيفي خمير منجمد سنگک و نان حاصل آز آن در قالب اندازه‌گيري درصد مخمر زنده‌مانده و افزايش حجم خمير پس از يخ‌زدايي و اندازه‌گيري دانسيته و سفتي بافت پس از پخت ارزيابي گرديد. یافته‌ها: نتايج نشان مي‌دهد، تنش انجماد و نگهداری طولانی خمیر منجمد باعث کاهش قابل ملاحظه‌ای در درصد مخمر‌های زنده مانده و حجم خمیر و هم‌چنین افزایش دانسیته و سفتی نان سنگک حاصل می‌شود.30 دقیقه پیش تخمیر باعث کاهش کیفیت خمیر منجمد و نان سنگک حاصل از آن شده است. نتیجه‌گیری: بنابراين کیفیت خمیر منجمد و نان حاصل از آن با انجماد و نگهداری منجمد به طور قابل توجهی تحت تأثیر قرار مي‌گيرد.

Title: حذف تولوئن از هوا با استفاده از خاصیت فوتوکاتالیستی نانوذرات دی اکسید تیتانیم پوشش داده شده بر روی فوم فلزی نیکل

http://hsr.mui.ac.ir/index.php/jhsr/article/view/1610

مقدمه: انتشار ترکیبات آلی فرار در هوای محیط‌های صنعتی و شهری سبب اثرات نامطلوبی بر سلامت انسان و محیط زیست می‌شود. هدف از انجام این مطالعه، حذف تولوئن از هوا، به عنوان بارزترین آلاینده در بین VOCها، با استفاده از فناوری فوتوکاتالیستی نانوذرات TiO2 پوشش داده شده بر روی فوم نیکل بود. روش‌ها: با استفاده از فرایند سل ژل، نانوذرات TiO2 تهیه گردید و سپس بر روی فوم فلزی نیکل پوشش داده شد. با استفاده از آزمایشات XRD و SEM مشخصات مرفولوژی و زیرساختاری نانوذرات تعیین گردید. تبدیل فوتوکاتالیستی تولوئن در فاز گازی تحت تابش نور فرابنفش با تزریق نمونه از راکتور استاتیک به GC-FID اندازه گیری شد. یافته‌ها: در این مطالعه ابتدا مشخصات فازی و ساختاری فوتوکاتالیست، تعیین گردید و سطح بهینه رطوبت نسبی برای انجام آزمایش، 30% تعیین شد. سپس، مقدار جذب سطحی تولوئن حدود 55% به‌دست آمد. در نهایت، پس از ثابت شدن غلظت تولوئن در راکتور، با استفاده از فعالیت فوتوکاتالیستی نمونه 55% از مقدار غلظت اولیه آن حذف شد. نتیجه‌گیری: با استفاده از نانوذرات TiO2 پوشش داده شده بر روی فوم نیکل، در دو مرحله جذب سطحی و تبدیل فوتوکاتالیستی بیش از 80% تولوئن حذف گردید، به طوری که غلظت تولوئن از ppm 100 به ppm 20 رسید.‏

Title: عوامل پیش‌بینی‌کننده مصرف مواد روان‌گردان در بین نوجوانان و جوانان شهر کرمانشاه با بهره‌گیری از تئوری رفتار برنامه‌ریزی شده

http://hsr.mui.ac.ir/index.php/jhsr/article/view/1682

مقدمه: در دو دهه اخیر مصرف داروهای روان‌گردان به خصوص در بین نوجوانان و جوانان رایج شده است، اين داروها در رديف دوم مصرف انواع مواد در دنيا قرار دارند. مطالعه حاضر با هدف تعیین عوامل پیش‌بینی کننده مصرف مواد روان‌گردان در میان جوانان و نوجوانان شهر کرمانشاه با بهره‌گیری از تئوري رفتار برنامه‌ریزی شده انجام شد. روش‌ها: این مطالعه توصيفي مقطعي در میان 385 نفر از نوجوانان و جوانان شهر کرمانشاه انجام گرفت. آزمودنی‌ها به روش نمونه‌گيري تصادفی ساده انتخاب شدند. پرسش‌نامه‌ شامل سه بخش كلي اطلاعات جمعیت‌شناختی، سؤالات آگاهی از پیامدهای مصرف مواد روان‌گردان و پرسش‌نامه سازه‌های تئوري رفتار برنامه‌ریزی شده بود، که به صورت خودگزارش دهی توسط شرکت‌کنندگان تكميل گرديد. داده‌های جمع‌آوري شده با نرم‌افزار SPSS-21 و بهره‌گیری از آزمون‌های رگرسیون و همبستگی پیرسون تجزیه و تحلیل گرديد. یافته‌ها: 2/7 و 2/4 درصد از شرکت‌کنندگان به ترتیب تجربه مصرف شیشه و اکستازی داشتند. نرم‌های انتزاعی (455/1=OR)، کنترل رفتار درک شده (793/0=OR) و قصد رفتار (440/1=OR) پيش‌بيني‌کننده‌های مصرف مواد روان‌گردان بودند. نتیجه‌گیری: به نظر می‌رسد مصرف مواد توسط دوستان نزدیک و به دنبال آن فشارهای همسالان به عنوان عامل ترغیب‌کننده مصرف، شرایط را برای مصرف مواد روان‌گردان فراهم می‌کند.

Title: بررسی رابطه نگرش ،باور های هنجاری وباورهای کنترلی وخودکارآمدی دانشجویان نسبت به غذای آماده (فست فود) با شیوه های دموگرافیکی در دانشجویان علوم پزشکی شهر همدان

http://hsr.mui.ac.ir/index.php/jhsr/article/view/1923

مقدمه: امروزه استفاده از غذای آماده درجوانان یک مشکل بهداشتی در ایران و سایر کشورهای جهان می‌باشد و نزدیک به یک سوم از اين افراد به طور روزانه از غذای آماده بیرون از منزل استفاده می‌کنند. این مطالعه با هدف بررسی وضعيت نگرش، باورهای هنجاری و باورهای کنترلی وخودکارامدی دانشجویان نسبت به مصرف غذای آماده در دانشجویان دانشگاه علوم پزشکی شهر همدان انجام شده است. روش‌ها: این پژوهش یک مطالعه توصیفی– تحلیلی و از نوع مقطعی مي‌باشد كه در بین 250 نفر از دانشجویان دانشگاه علوم پزشکی همدان در سال91 اجرا شد. دانشجویان دانشگاه علوم پزشکی همدان پرسش‌نامه محقق ساخته كه روايي و پايايي آن مورد تأييد قرار گرفت را تكميل كردند. اطلاعات جمع‌آوری شده و با استفاده از نرم‌افزار آماری 16spss مورد تجزیه و تحلیل قرار گرفت. یافته‌ها: نگرش با متغیرهای داشتن رژیم غذایی کاهش وزن و محل سکونت رابطه معنی‌داری داشت. باورهای هنجاری تنها با متغیرهای جنسیت و مقطع تحصیلی رابطه معني‌داری داشت و سطح خود کارامدی دانشجویان نسبت به غذای آماده با متغیرهای سن و جنسیت رابطه معنی‌‌داری داشت. در واقع در جنس مؤنث خود کارامدی بیشتری در رابطه با مصرف غذای آماده وجود دارد. باورهای کنترلی با هیچ یک از متغیرهای مورد بررسی رابطه معنی‌داری نداشت. نتیجه‌گیری: اطلاعات به‌دست آمده از این مطالعه حاکی از آن است که مصرف غذای آماده در بین پسران و هم‌چنین در بین دانشجویان ساکن خوابگاه بیشتر می‌باشد که این خود بیان‌گر نیاز به ایجاد نگرش و باورهای صحیح در رابطه با مصرف غذای آماده در بین دانشجویان می‌باشد

Title: طراحی مدل ریاضی ارزیابی عملکرد بیمارستان ها با رویکرد تلفیقی ‏FHNBSC‏ ، ‏SAW‏ و ‏LINMAP

http://hsr.mui.ac.ir/index.php/jhsr/article/view/2017

مقدمه: نظر به اهميت استقرار يك نظام ارزيابي عملكرد مناسب و راهبردي در جهت دستيابي به راهبردهای ‏سازمان‌ها، ‏‏کارت امتیازی متوازن نوعی مدل ارزیابی و سنجش یکپارچه عملکرد می‌باشد که چارچوبی برای تبدیل رسالت، ‏چشم‌انداز و راهبرد به مجموعه‌ای از اهداف و سنجه‌های عملکرد در زیرمجموعه مناظر مشتری، ‏فرایند‌های داخلی، یادگیری– رشد و مالی را فراهم می‌آورد. روش‌ها: در اين پژوهش براي برطرف نمودن نقايص اين مدل در ارزیابی عملکرد بیمارستان‌ها، از تلفيق تكنيك‌هاي ‏تحليل‎ ‎شبكه‌اي فازی، مجموع وزین ساده و لینمپ بهره گرفته شده است. یافته‌ها: در این پژوهش براساس برنامه‌ی راهبردی بیمارستان‌های دولتی و کارت سلسله مراتبی امتیازی متوازن، ‏مهم‌ترین معیارها در قالب چهار‌گانه کارت امتیازی متوازن استخراج گردیدند. بعد از شناسایی معیارها و تشکیل ‏درخت سلسله مراتبی نقشه راهبردی، با استفاده از نظرات خبرگان وابستگی بین قسمت‌های مختلف این درخت ‏سلسله مراتبی معین و همراه با ماتریس مقایسات زوجی، ‏FANP‏ تشکیل و وزن هرکدام از معیارها مشخص ‏گردید. نتیجه‌گیری: پنج بیمارستان دولتی یزد به عنوان نمونه تجربی انتخاب گردیدند. ابتدا با استفاده از برنامه راهبردی و ‏HBSC‏ ‏مهم‌ترین معیارها استخراج گردیدند و بر اساس درخت سلسله مراتبی نقشه راهبردی وزن نهایی شاخص‌ها با ‏استفاده از تحلیل شبکه‌ای فازی صورت پذیرفت (‏FNHBSC‏). در این مرحله با استفاده از تکنیک ‏SAW، رتبه‌‏بندی اولیه و در نهایت با استفاده از تکنیک ‏LINMAP‏ رتبه‌بندی نهایی بیمارستان‌ها صورت پذیرفت. در نهایت بر ‏اساس یافته‌های پژوهش و مبتنی بر ‏نظرات خبرگان، مدل پیشنهادی قابلیت و کارایی لازم جهت ‏ارزیابی عملکرد ‏و رتبه‌بندی بیمارستان های ‏دولتی را دارا می‌باشد.‏

Title: بررسی تأثير آموزش زوج‌درماني شناختي رفتاري اسلام‌محور بر تعهد زناشويي زنان

http://hsr.mui.ac.ir/index.php/jhsr/article/view/1182

مقدمه: تعهد زناشويي بين همسران يك پيش‌‌بیني‌كننده‌ي مهم براي ازدواج‌هاي رضايت‌بخش و پايدار است، تعهد مادام‌العمر به ازدواج، وفاداري نسبت به همسر خويش، ارزش‌هاي اخلاقي قوي، احترام نسبت به همسر خويش به عنوان بهترين دوست و تعهد نسبت به وفاداري جنسي از ويژگی‌هاي ازدواج‌هاي رضايت‌بخش است. هدف اين پژوهش تعيين ميزان اثربخشي آموزش زوج‌درماني شناختي رفتاري اسلام ‌محور بر تعهد زناشويي زنان شهر قم بوده است. روش‌ها: اين پژوهش نيمه‌تجربي است و در آن از پيش‌آزمون و پس‌آزمون با گروه کنترل استفاده شده است. جامعه پژوهش شامل آن دسته از زوجين مراجعه‌کننده به مرکز مشاوره خانواده در سال 1390-1391 مي‌باشد که به صورت خود معرف مراجعه کرده بودند. نمونه پژوهش شامل 100 نفر از زنانی است که داوطلب به شرکت در مطالعه بودند، به صورت نمونه در دسترس و تصادفي در گروه مداخله و کنترل قرار گرفتند. به نمونه آزمایش به صورت گروهی آموزش شناختی رفتاری اسلام‌محور داده شد را ابزار پژوهش پرسش‌نامه دموگرافیک و پرسش‌نامه تعهد زناشويي (DCI یا Dimensions of Commitment Inventory) Adams & Jons مورد استفاده قرار گرفت. براي تجزيه و تحليل داده‌ها از نرم‌افزار 16-SPSS  با استفاده از روش‌هاي آمار توصيفي و تحليلی (کوواريانس) با سطح معنی‌داری 05/0 استفاده شد. یافته‌ها: نتايج نشان مي‌دهد که آموزش زوج‌درماني اسلام‌محور، تعهد زناشويي زوجين و ابعاد آن، يعني تعهد شخصي، اخلاقي و ساختاري زوجين را افزايش داده است (001/0>P). نتیجه‌گیری: با توجه به نقش مذهب در زندگی زناشویی، این رویکرد می‌تواند در سایر ابعاد دیگر مانند رضایت و سازگاری و ... که آنان نیز مستقیم و غیرمستقیم بر تعهد زناشویی اثربخش هستند، مؤثر باشد.

Title: ارتباط خودکارامدی با سازه‌های مدل اعتقاد بهداشتی در بین بیماران دیابتی نوع 2 مراجعه‌کننده به انجمن دیابت ایران

http://hsr.mui.ac.ir/index.php/jhsr/article/view/1545

مقدمه: امروزه افزایش تعداد مبتلايان به ديابت در دنيا این بیماری را به بزرگ‌ترين اپيدمي جهاني تبديل نموده كه خودکارا‌مدی بیمار یکی از عناصر کلیدی در پیشگیری و درمان دیابت محسوب می‌شود. لذا مطالعه حاضر با هدف تعیین ارتباط خودکارامدی با سایر سازه‌های مدل اعتقاد بهداشتی در بین بیماران دیابتی نوع 2 انجام گردید. روش‌ها: اين پژوهش يك مطالعه مقطعی است كه80 نفر بيماران مبتلا به ديابت نوع 2 مراجعه‌كننده به انجمن ديابت ايران بودند كه در طول 2 ماه در سال1392، به شيوه نمونه‌گيری غير تصادفی در دسترس انتخاب شدند. ابزار گردآوری اطلاعات پرسش‌نامه دو بخشی اطلاعات دموگرافیک (شامل 9 سؤال)وابزار پرسش‌نامه محقق ساخته در خصوص بيماري ديابت مبتني بر سازه‌هاي مدل اعتقاد بهداشتي (51 سؤال) بود. داده‌های جمع‌آوری شده با استفاده از نرم‌افزار SPSS نسخه 18 مورد تجزیه و تحلیل قرار گرفت. یافته‌ها: میانگین سنی شرکت‌کنندگان در مطالعه 89/8 ± 16/55 سال بود. بین سازه‌های تهدید درك‌شده، منافع درك شده، موانع درك شده، راهنما براي عمل با خودکارامدی همبستگی آماری معنی‌داری و مثبتی مشاهده شد (05/0P<) . در بین سازه‌های مختلف، موانع درک شده بیشترین همبستگی را بر میزان خودکارامدی بیماران داشت (001/0p<، 98/0r=). نتیجه‌گیری: بر اساس نتايج به‌دست آمده مداخلات آموزش و ارتقاء سلامت می‌تواند با ارتقاء توانايي‌های بیماران دیابتی جهت غلبه بر موانع اعتماد به نفس و احساس ارزشمندی آنان را بهبود بخشد و این رویکرد به عنوان گامي‌ ضرور‌ي‌ جهت‌ ارتقاء خودکارامدی بیماران پیشنهاد می‌گردد.

Title: رابطه شاخص‌های سلامت سازمانی و اثربخشی سازمان در نظام سلامت

http://hsr.mui.ac.ir/index.php/jhsr/article/view/1781

مقدمه: سلامت سازمان باعث اعتماد بیشتر کارکنان به سازمان و افزایش انگیزش و علاقه‌مندی به کار و بالطبع باعث افزایش کارایی آنان می شود و لذا هدف اصلی تمام سازمان‌ها ارتقا اثربخشی و کارایی می‌باشد. از اين رو، پژوهش حاضر با هدف بررسي رابطه شاخص‌های سلامت سازمانی و اثربخشی سازمان در بین کارکنان دانشگاه علوم پزشکی اصفهان صورت گرفت. روش‌ها: مطالعه حاضر از نوع توصیفی- همبستگی است که در سال 1392 انجام شد. جامعه پژوهش، متشکل از کارکنان شاغل در هفت معاونت ستادی دانشگاه علوم پزشکی اصفهان به تعداد 672 نفر بوده که به عنوان حجم نمونه  به روش طبقه‌ای- تصادفی و برابر 185 نفر انتخاب شد. داده‌ها با استفاده از پرسش‌نامه‌های سلامت سازمانی هوی و فیلدمن و پرسش‌نامه اثربخشی سازمان پارسونز سنجیده و جمع‌آوری شد و برای تجزیه و تحلیل داده ها نیز از ضریب همبستگی پیرسون در نرم‌افزار SPSS-18 استفاده شد. یافته‌ها: نتایج نشان داد که میانگین سلامت سازمانی به طور کلی از دیدگاه کارکنان 86/11 ± 66/164 و میانگین میزان اثربخشی سازمان به طور کلی از دیدگاه کارکنان 22/8 ± 66/98 بوده هم‌چنین در سطح نهادی مؤلفه‌ی یگانگی نهادی، در سطح فنی مؤلفه‌ی روحیه و در سطح اداری مؤلفه‌های نفوذ مدیر، ملاحظه‌گری رابطه قوی، مستقیم و معنی‌داری با اثربخشی سازمان داشته‌اند (05/0P-Value<). نتیجه‌گیری: نتایج مطالعه حاضر نشان داد که سلامت سازمانی و ابعاد آن تا حد زیادی اثربخشی را تحت‌تأثیر خود قرار می‌دهند.

Title: تحلیل مدل‌های رگرسیون شمارشی با صفر انباشته به روش بیز و بکارگیری آن‌ها در شناسایی عوامل مؤثر بر تعداد دفعات اهدای مجدد خون

http://hsr.mui.ac.ir/index.php/jhsr/article/view/2166

مقدمه: هدف اصلی مراکز انتقال خون، تأمین خون سالم و کافی بر روی بیماران می‌باشد. برای آماده کردن خون سالم و کافی جهت تأمین نیاز بیماران، آگاهی از عواملی که افراد را به اهدای خون دعوت می‌کند و از خروج آن‌ها از چرخه اهدا جلوگیری می‌کند امری ضروری است. با توجه به اهمیت اهداکنندگان خون به‌عنوان تنها منبع خون، در این مطالعه به بررسی عوامل مؤثر بر تعداد دفعات اهدای مجدد خون اهداکنندگان شهرکرد بر اساس مدل‌های رگرسیون شمارشی با صفر انباشته پرداخته شده است. روش‌ها: مطالعه از نوع توصیفی تحلیلی است که بر روی داده‌های اهدای خون یک نمونه تصادفی به حجم 864 نفر از اهداکنندگان خون بار اول پایگاه انتقال خون شهرکرد انجام گرفت. متغیر پاسخ برابر با تعداد اهدای خون طی پنج سال و متغیرهای مستقل شامل سن، شغل، جنس، و وزن هستند. تحلیل داده‌ها بر اساس مدل‌های رگرسیون شمارشی با صفر انباشته شامل مدل رگرسیون پواسون با صفر انباشته، دوجمله‌ای منفی با صفر انباشته و پواسون تعمیم یافته با صفر انباشته انجام گرفت. برآورد پارامترها به روش بیزی و بر اساس الگوریتم مونت کارلوی زنجیر مارکوفی و با استفاده از نرم‌افزار Win BUGS انجام شد. مقایسه مدل‌ها بر اساس معیار بیزی اطلاع انحرافی انجام شد. یافته‌ها: مدل رگرسیون پواسون تعمیم یافته با صفر انباشته با داشتن کمترین مقدار معیار اطلاع انحرافی نسبت با سایر مدل‌ها، به عنوان بهترین مدل رگرسیون شمارشی با صفر انباشته برای داده‌ها انتخاب شد. متغیر وزن تأثیر معنی‌دار بر تعداد دفعات اهدای مجدد داشت به‌طوری‌که با افزایش وزن اهداکنندگان، تعداد دفعات اهدای مجدد خون افزایش یافته است. هم‌چنین تعداد دفعات اهدای مجدد خون ارتباط معکوس با سن داوطلبان اهدای خون داشت. نتیجه‌گیری: تعداد دفعات اهدای مجدد خون با وزن ارتباط مستقیم و با سن ارتباط معکوس داشت. لذا لازم است با فرهنگ‌‌سازی و آموزش عمومی، افراد با سن بالا و با وزن متوسط را به اهدای خون تشویق کنیم.

Title: طراحی سیستم تشخیصی هوشمند به‌منظور پیش‌بینی چاقی در کودکان با استفاده از عوامل شیوه زندگی، وضعیت اجتماعی- اقتصادی و اطلاعات وراثتی

http://hsr.mui.ac.ir/index.php/jhsr/article/view/2161

مقدمه: چاقی یکی از مهم‌ترین مشکلات تغذیه‌ای در سراسر دنیا است. اگرچه مطالعات به ‌غیر از مشکلات فیزیکی و حرکتی، گزارشی مبنی بر بروز بیماری‌های مزمن ناشی از چاقی در دوران کودکی ارایه نداده‌اند؛ اما چاقی در کودکی می‌تواند منجر به طیف وسیعی از مشکلات در آینده شود. در این مطالعه با هدف تشخیص زود هنگام بر آن هستیم تا با طراحی یک سیستم هوشمند احتمال وقوع چاقی را بر اساس اطلاعات اولیه‌ای بر اساس نحوه شیوه زندگی و یا سایر متغیرهای عمومی اولیه، پیش‌بینی کنیم. روش‌ها: در این مطالعه 9795 نفر (17/49% پسر) درگروه‌های سنی 6 تا 18 سال براساس چهارمین فاز مطالعه ملی گسترده کاسپین، مورد بررسی قرارگرفته‌اند. متغیرهای ورودی سیستم براساس عادات تغذیه‌ای، فعالیت ورزشی، اطلاعات وراثتی، وضعیت اجتماعی و اقتصادی و سابقه چاقی و دیابت مشخص گردیده‌اند. سپس بر اساس روش‌های داده‌کاوی و هوش مصنوعی، مشکل چاقی شناسایی و بررسی شده‌ است. روش‌های انتخاب ویژگی نیز برای بهینه‌سازی سیستم طراحی ‌شده در نظر گرفته ‌شده است. یافته‌ها: عملکرد روش‌های دسته‌بندی مورد مطالعه توسط روش ارزیابی متقابل دولایه‌ای مورد بررسی قرار گرفت. با توجه به نتایج حاصل از ارزیابی، بهترین نتیجه توسط روش دسته‌بندی ماشین‌بردار پشتیبان به‌ دست آمده است. دقت و صحت این روش شناسایی پس از انتخاب ویژگی به ترتیب 3/63 و 7/83 درصد بوده است. ویژگی‌هایی ازقبیل سن، فعالیت ورزشی، نوع تغذیه در دوران نوزادی و سابقه دیابت در خانواده به‌عنوان تأثیرگذارترین ویژگی‌ها در ایجاد روند چاقی در دو گروه دختر و پسر شناسایی ‌شده‌اند. نتیجه‌گیری: طراحی سیستم‌های تشخیصی هوشمند با استفاده از پارامترهایی از قبیل شیوه زندگی، وضعیت اجتماعی اقتصادی و اطلاعات وراثتی می‌تواند در پیشگیری چاقی کودکان در آینده کمک نموده و به اصلاح شیوه زندگی آنان، بیانجامد. طراحی این سیستم‌های هوشمند تحت وب، می‌تواند خدمات پیشگیری را برای خانواده‌ها به‌ راحتی ممکن سازد..

Title: بررسی تأثیر فعالیت بدنی بر ناراحتی‌های اسکلتی عضلانی در شاغلین یکی از صنایع دستی

http://hsr.mui.ac.ir/index.php/jhsr/article/view/1630

مقدمه: علی‌رغم وجود دانش گسترده برای ناراحتی‌های اسکلتی عضلانی مرتبط با کار و فاکتورهای مختلفی که باعث شدت این ناراحتی‌ها می‌گردند، در‌مورد تأثیر ورزش به‌ عنوان یک عامل پیش‌گیرنده اطلاعات کمی وجود دارد. صنایع‌دستی از مشاغل پر‌مخاطره از این نظر محسوب می‌گردد که وظایفی با ماهیت تکراری و پوسچرهای نامطلوب را به فرد تحمیل می‌کند. انجام مداخلات اصلاحی مثل فعالیت‌های ورزشی برای این افراد از ضروریات است. این مطالعه با هدف بررسی تأثیر فعالیت‌های بدنی بر‌روی این ناراحتی‌ها در بین شاغلین یکی از صنایع‌‌دستی انجام گرفت. روش‌ها: این مطالعه، یک مطالعه توصیفی‌- تحلیلی، به‌صورت مقطعی (cross-sectional)، در 100 نفر از شاغلین یکی از صنایع‌دستی انجام گرفت. نمونه‌گیری به‌صورت سرشماری و از کلیه کارگاه‌های منطقه انجام شد. اطلاعات مربوط به مشخصات فردی و فعالیت‌های بدنی افراد توسط فرم دموگرافیک و ناراحتی‌های اسکلتی عضلانی شاغلین با‌ استفاده از پرسش‌نامه کرنل انجام گرفت. آنالیز داده‌ها با استفاده از آزمون آماری Chi-square، Independent-t و آنالیز واریانس یک‌طرفه انجام شد و سپس با استفاده از نرم‌افزار SPSS نسخه 18 ارزیابی گردید. یافته‌ها: بالاترین شکایات افراد، مربوط به ناحیه شانه راست (36%)،  مچ دست راست (26%)، گردن (25%) و قسمت فوقانی بازوی راست (24%) بوده است. بین ناراحتی اسکلتی عضلانی ناحیه‌ی مچ دست چپ، قسمت تحتانی پشت، گردن و انجام فعالیت‌های ورزشی ارتباط معنی‌دار وجود دارد. نتیجه‌گیری: بر‌اساس نتایج به‌دست آمده، فعالیت‌های ورزشی منظم، اما نه خیلی سنگین می‌تواند تأثیر مثبت بر‌روی کاهش ناراحتی‌های اسکلتی عضلانی بگذارد.

Title: ارزیابی میزان مواجهه با گرما در کارگران نانوایی‌های شهر اصفهان با کاربرد شاخص دمای تر گویسان، شاخص نمره استرین گرمایی و دمای دهانی

http://hsr.mui.ac.ir/index.php/jhsr/article/view/1884

مقدمه: گرما یکی از مهم‌ترین عوامل زیان‌آور بسیاری از محیط‌های کاری از جمله نانوایی‌ها است که می‌تواند موجب بروز بیماری‌های مرتبط با گرما و کاهش بهره‌وری می‌شود. این مطالعه با هدف ارزیابی مواجهه کارگران شاغل در نانوایی‌های شهر اصفهان با تنش‌های حرارتی محیط کار خود از طریق بررسی ارتباط بین شاخص دمای تر گویسان (WBGT یا Wet Bulb Globe Temperature) ، شاخص نمره‌گذاری استرین گرمایی (HSSI یا Heat Strain Scoring Index) و دمای دهانی انجام شد. روش‌ها: این مطالعه مقطعی در ماه‌های اردیبهشت تا تیر سال 1392 روی 82 نفر از کارگران شاغل در 43 واحد نانوایی شهر اصفهان انجام شد. جهت ارزیابی استرس گرمایی محیط کار از شاخص دمای تر گویسان (WBGT) و برای بررسی استرین گرمایی از دمای دهانی و شاخص نمره استرین گرمایی (HSSI) استفاده گردید. جهت بررسی ارتباط بین شاخص دمای تر گویسان، شاخص نمره‌گذاری استرین گرمایی و دمای عمقی نیز از آزمون ضریب همبستگی پیرسون استفاده شد. یافته‌ها: کارگران نانوایی در معرض استرس‌های حرارتی محیط کار خود قرار داشتند. بیشتر از 50 درصد افراد مورد بررسی دچار استرین گرمایی بودند. هم‌چنین میزان استرس گرمایی در نانوایی‌های با تکنولوژی پخت سنتی بیشتر از نانوایی‌های با تکنولوژی پخت ماشینی بود به طوری که بیشتر از 68 درصد افراد شاغل در نانوایی‌های سنتی مواجهه غیر مجاز با استرس حرارتی داشتند. نتایج نشان داد که ضریب همبستگی پیرسون بین شاخص دمای تر گویسان و شاخص نمره استرین گرمایی بالاتر از 62/0 بود. نتیجه‌گیری: : بر اساس نتایج این مطالعه وضعیت استرس گرمایی در نانوایی‌های شهر اصفهان نگران‌کننده بود. به نظر می‌رسد برنامه‌ریزی در جهت کنترل استرس‌های گرمایی در نانوایی‌های شهر اصفهان ضروری باشد. در این پژوهش شاخص نمره استرین گرمایی رابطه معنی‌دار و مستقیمی با شاخص دمای تر گویسان نشان داد لذا به نظر می‌رسد بتوان از شاخص نمره استرین گرمایی در ارزیابی استرین حرارتی محیط‌های گرم مانند نانوایی‌ها استفاده کرد.

Title: مقایسه کارایی صافی ماسه‌ای کند متداول با صافی اصلاح شده با بستر شیشه بازیافتی خرد شده در حذف کدورت و کلیفرم‌های کل و مدفوعی از منابع آب

http://hsr.mui.ac.ir/index.php/jhsr/article/view/1933

مقدمه: هدف از مطالعه حاضر مقایسه کارایی صافی ماسه‌ای کند متداول با صافی اصلاح شده با بستر شیشه بازیافتی خردشده در حذف کدورت و کلیفرم‌های کل و مدفوعی از منابع آب بود. روش‌ها: در این مطالعه به مدت 4 ماه کارایی صافی های ماسه‌ای کند متداول (صافی نوع 1) و صافی اصلاح شده با بستر شیشه بازیافتی خردشده (صافی نوع 2) در نمونه پایلوت ساخته شده مقایسه شد. آب مورد استفاده، آب چاه بود که مقادیر مناسب کدورت و کلیفرم به‌صورت سنتتیک به آن اضافه شد. در این مطالعه، کارایی حذف کدروت، و کلیفرم‌های کل و مدفوعی مورد بررسی قرار گرفت. به منظور تعیین مشخصات آب ورودی و خروجی از صافی‌ها و مقایسه با رهنمودهای موجود، سایر پارامترهای فیزیکوشیمیایی آب نیز مورد آنالیز قرار گرفت. یافته‌ها: میزان حذف کدورت توسط صافی نوع 1 و صافی نوع 2 به ترتیب 28/97 و 93/96 درصد و میانگین کدورت خروجی نیز به ترتیب 19/0 ± 49/0 و NTU 36/0 ± 55/0 بودند. میزان حذف کلیفرم کل و کلیفرم مدفوعی برای صافی نوع 1 به ترتیب 89/2 وlog  29/2 و برای صافی نوع 2 به ترتیب 62/2 و  log46/2 به‌دست آمد. هر دو صافی تأثیر چندانی بر سایر پارامترهای فیزیکوشیمیایی نداشتند، و مقادیر این پارامترها در آب تصفیه شده زیر حد استاندارد WHO و 1053 ایران بود. نتیجه‌گیری: کارایی صافی نوع 2 در حذف پارامترهای میکروبی و فیزیکوشیمیایی تقریباً مشابه صافی نوع 1 بود. هم‌چنین بستر شیشه بازیافتی در مقایسه با بستر ماسه‌ای متداول دارای افت فشار کمتر، مقرون به صرفه و دوستدار محیط زیست است و می‌تواند جایگزین مناسبی برای صافی ماسه‌ای کند متداول باشد.

Title: بررسی میزان دریافت روزانه مواد مغذی حاوی حاوی آنتی اکسیدان و ارتباط آن با وقوع پره اکلامپسی در زنان باردار شهر تهران در سال 1390

http://hsr.mui.ac.ir/index.php/jhsr/article/view/1142

مقدمه: با توجه به اینکه پره اکلامپسی یک عارضه بالقوه خطرناک و کشنده در بارداری است این پژوهش با هدف تعیین میزان دریافت روزانه مواد مغذی آنتی‌اکسیدان و ارتباط آن با وقوع پره‌اکلامپسی در زنان باردار شهر تهران در سال 1390 انجام شد. روش‌ها: این مطالعه طولی (آینده‌نگر) میان 1033 مادر باردار که برای مراقبت‌های قبل از بارداری به درمانگاه‌های سطح شهر تهران مراجعه می‌کردند انجام شد. در بدو ورود به مطالعه، برای تمام نمونه‌ها پرسش‌نامه بسامد خوراک و پرسش‌نامه دموگرافیک، یک بار قبل از هفته 20 از طریق مصاحبه چهره به چهره تکمیل گردید. اطلاعات تغذیه‌ای به دست آمده از طریق پرسش‌نامه هر واحد مورد پژوهش، که در آن مواد غذایی مصرف شده توسط مادر در طی یک ماه گذشته ثبت شده بود توسط پژوهشگر به گرم تبدیل شد و اطلاعات به‌دست آمده از پرسش‌نامه به نرم‌افزار N4 برای محاسبه مواد مغذی دریافتی مادر داده شد. مادران باردار از زمان ورود به مطالعه تا زمان زايمان تحت مراقبت و پيگيري قرار گرفتند. در صورت وجود فشارخون ≥90/140 ميلي‌متر جيوه و پروتئين‌اوري پس از هفته 20 بارداری تشخیص پره اکلامپسی برای مادر داده می‌شد. سطح معنی‌داری آزمون‌های به کار رفته کمتر از 05/0 بود. یافته‌ها: از 1033 مادر باردار 20  نفر (9/1%) مبتلا به پره‌اکلامپسی شدند. نتیجه آزمون T مستقل نشان داد که اختلاف معنی‌داری در میانگین دریافت روزانه مواد مغذی آنتی‌اکسیدان (روی، ویتامین A،C، E) در دو گروه مبتلا و غیر مبتلا به پره‌اکلامپسی وجود دارد. به‌طوری که میانگین سطح دریافتی این مواد در زنان مبتلا به پره‌اکلامپسی به طور معنی‌داری کمتر بود. نتیجه‌گیری: میزان دریافت روزانه مواد مغذی آنتی‌اکسیدان در این مطالعه کمتر از میزان توصیه شده بود. هم‌چنین نتایج نشان داد کاهش دریافت روزانه مواد مغذی آنتی‌اکسیدان با وقوع پره‌اکلامپسی ارتباط دارد.

Title: ارزیابی پتانسیل تجمع زیستی و میزان مواجهه با سرب و کادمیوم ناشی از مصرف میگوی ببری سبزدر شهر تهران در سال 1391

http://hsr.mui.ac.ir/index.php/jhsr/article/view/1844

مقدمه: کنترل ایمنی فراورده‎های شیلات از نظر تجمع فلزات سنگین یکی از موارد مهمی است که براي حفظ سلامت افراد جامعه و نیل به تضمین ایمنی این ماده غذایی ارزشمند باید مورد توجه قرار گیرد. هدف از این تحقیق، ارزیابی پتانسیل تجمع زیستی و میزان مواجهه با سرب و کادمیوم ناشی از مصرف میگوی ببری سبز در شهر تهران در سال 1391 می‌باشد. روش‌ها: نمونه‌های میگو به صورت تصادفی از چهار منطقه عمده عرضه آبزیان در مناطق جنوب، شمال، شرق و غرب شهر تهران در سال 1391 در دوره زمانی عرضه میگوی ببری سبز غیرمنجمد نمونه‌برداری شدند. پس از انجام عملیات آماده‌سازی و هضم قسمت‌های عضله و پوسته میگو، مقادیر فلزات سنگین سرب و کادمیوم توسط دستگاه طیف سنجی نشر اتمی پلاسمای جفت شده القایی اندازه‌گیری شد و میزان مواجهه مصرف‌کنندگان با این عناصر مورد ارزیابی قرار گرفت. یافته‌ها: نتایج نشان داد که میزان میانگین تجمع سرب در پوست و عضله میگو به ترتیب 59/1 ± 37/5 و 12/1 ± 59/5 و برای فلز کادمیوم به ترتیب 61/0 ± 11/1 و 62/0 ± 79/1 میکروگرم بر کیلوگرم می‌باشد. هم‌چنین میانگین غلظت سرب و کادمیم در عضله بیش از غلظت آن در پوست میگو مي‌باشد. اختلاف بین میانگین تجمع سرب و کادمیم در بخش خوراکی میگو معنی‎دار نیست. میزان مواجهه مصرف‌کنندگان با سرب و کادمیم ناشی از مصرف بافت خوراکی میگو به ترتیب یک پنجاهم و یک سی‎ام مقدار مجاز درنظر گرفته شده برای میزان دریافت روزانه موقتی می‌باشد. نتیجه‌گیری: ﻣﻘﺎﻳﺴﻪ میانگین غلظت سرب و کادمیم در بخش خوراکی میگو با استانداردهای ملی و بین‌المللی نشان می‌دهد که مقدار این عناصر کمتر از حد مجاز بوده و خطری برای سلامت مصرف‌کننده ندارد. نتایج مفید حاصل از این ارزیابی از سطح عرضه می‎تواند به عنوان الگویی جهت پایش سلامت میگو و سایر آبزیان مورد استفاده قرار گیرد.
